# Partial Peptide of α-Synuclein Modified with Small-Molecule Inhibitors Specifically Inhibits Amyloid Fibrillation of α-Synuclein

**DOI:** 10.3390/ijms14022590

**Published:** 2013-01-28

**Authors:** Wataru Yoshida, Natsuki Kobayashi, Yasuhiko Sasaki, Kazunori Ikebukuro, Koji Sode

**Affiliations:** Department of Biotechnology, Graduate School of Engineering, Tokyo University of Agriculture & Technology, 2-24-16 Naka-cho, Koganei, Tokyo 184-8588, Japan; E-Mails: wyoshida@cc.tuat.ac.jp (W.Y.); nkobayashi@umnpharma.com (N.K.); yasuhiko.sasaki@shino-test.co.jp (Y.S.); ikebu@cc.tuat.ac.jp (K.I.)

**Keywords:** α-synuclein, amyloid β, pyrroloquinoline quinone, Baicalein, EGCG

## Abstract

We have previously reported that pyrroloquinoline quinone (PQQ) prevents the amyloid formation of α-synuclein, amyloid β_1–42_ (Aβ_1–42_), and mouse prion protein. Moreover, PQQ-modified α-synuclein and a proteolytic fragment of the PQQ-modified α-synuclein are able to inhibit the amyloid formation of α-synuclein. Here, we identified the peptide sequences that play an important role as PQQ-modified specific peptide inhibitors of α-synuclein. We demonstrate that the PQQ-modified α-Syn_36–46_ peptide, which is a partial sequence of α-synuclein, prevented α-synuclein amyloid fibril formation but did not inhibit Aβ_1–42_ fibril formation. In addition, the α-synuclein partial peptide modified with other small-molecule inhibitors, Baicalein and epigallocatechin gallate (EGCG), prevented α-synuclein fibril formation. Currently reported quinone amyloid inhibitors do not have selectivity toward protein molecules. Therefore, our achievements provide a novel strategy for the development of targeted specific amyloid formation inhibitors: the combination of quinone compounds with specific peptide sequence from target proteins involved in amyloid formation.

## 1. Introduction

Conformational neurodegenerative diseases are characterized by the formation and accumulation of misfolded proteins or amyloid fibrils. Abnormal aggregation of α-synuclein (α-Syn), Amyloid β (Aβ), and prion protein are associated with the pathogenesis of Parkinson’s disease (PD), Alzheimer’s disease, and prion disease, respectively. Oligomers and/or amyloid fibrils composed of α-Syn, Aβ, and prion protein are considered to be cytotoxic. Therefore, agents that inhibit oligomerization and/or amyloid fibril formation could be useful for development of novel therapies [1–7]. Several small compounds have been identified that prevent fibril formation by amyloid-forming proteins [8–19]. Some of these compounds have a quinone structure, such as Baicalein [11] and epigallocatechin gallate (EGCG) [15], and covalently bind to the Lys residues of the amyloid-forming protein *via* Schiff-base formation. It has been reported that EGCG binds to protein *via* Schiff-base formation through autoxidation [20]. We have reported that pyrroloquinoline quinone (PQQ) prevents the amyloid fibril formation of α-Syn, Aβ_1–42_ and mouse prion protein *in vitro* [21,22]. Since the Schiff-base formation of these quinone compounds does not have selectivity towards protein molecules, non-specific interaction of these quinone compounds with amine groups will occur *in vivo*, which may consequently result in low therapeutic efficacy.

We have also reported that PQQ-modified α-Syn prevents the amyloid formation of intact α-Syn [21]. We assumed that PQQ-modified α-Syn would specifically bind to intact α-Syn to prevent amyloid formation. We recently reported that proteolytic-digested PQQ-modified α-Syn inhibits amyloid formation as effectively as the PQQ-modified α-Syn does [23]. These results indicate that PQQ-modified α-Syn partial peptide inhibits amyloid formation by interacting with intact α-Syn, based on the specific molecular recognition. The mechanism of amyloid fibril formation is based on the formation of core structure mediated by sequence-specific interaction among α-Syn monomers. Therefore, PQQ-modified α-Syn partial peptides will specifically inhibit α-Syn amyloid formation.

In order to evaluate the potential application of PQQ-modified α-Syn partial peptide as a specific inhibitor for α-Syn amyloid formation, we first identified the peptide sequence of the PQQ-modified α-Syn partial peptide, which inhibits the amyloid formation. Based on the identified sequence information, we prepared the PQQ-modified α-Syn partial peptides, and investigated their ability to block amyloid formation inhibition using α-Syn and Aβ_1–42_. In addition to PQQ, the α-Syn partial peptide was modified using other small molecule inhibitors, Baicalein and EGCG, and their inhibitory effects on α-Syn fibril formation were evaluated.

## 2. Results and Discussion

### 2.1. Identification of Proteolytic-Digested α-Synuclein Partial Peptide Sequence

We previously reported the preparation of a PQQ-modified α-Syn partial peptide using reverse-phase chromatograms [23]. The reverse-phase chromatograms for proteolytic products of unmodified α-Syn were almost identical with those of the chromatograms for the proteolytic products of PQQ-modified α-Syn. Thus, we analyzed peptide sequences of proteolytic products of unmodified α-Syn in the fraction, which correspond to Sp-7 by LC/MS (Figure S1). We identified five peptide sequences corresponding to 3–13, 21–28, 36–46, 47–57 and 127–131 residues of the primary structure of α-Syn (Table 1). Among these, 3 peptides corresponding to 3–13, 21–28, and 36–46 residues (α-Syn_3–13_, α-Syn_21–28_ and α-Syn_36–46_, respectively) have Lys residues, which are capable of binding to PQQ *via* Schiff-base formation. These results suggest that the three peptides would interact with intact α-Syn to inhibit the amyloid formation by PQQ modification.

### 2.2. Evaluation of Inhibitory Effects of PQQ-Modified Peptides on Amyloid Formation of α-Synuclein

We synthesized the three peptides (α-Syn_3–13_, α-Syn_21–28_ and α-Syn_36–46_) and modified them with PQQ by co-incubation for seven days. The PQQ-modified peptides were purified by size exclusion chromatography. PQQ-modified α-Syn has absorbance at 420 nm [21], and the intact peptides have absorbance at both 210 nm and 280 nm. In size exclusion chromatography separation, each PQQ modified peptide sample showed two peaks when the elution was monitored by determining the absorbance at both 210 nm and 280 nm (Figure S2). We observed large absorbance at 420 nm in the first peaks, which is the characteristic absorbance of PQQ, indicating that the first peaks contain PQQ-modified peptides. Thus, the first peaks were collected to evaluate the inhibitory activity. The collected PQQ-modified α-Syn_3–13_, α-Syn_21–28_ and α-Syn_36–46_ were designated as α-Syn_3–13_-PQQ, α-Syn_21–28_-PQQ and α-Syn_36–46_-PQQ, respectively.

**Figure 1:**
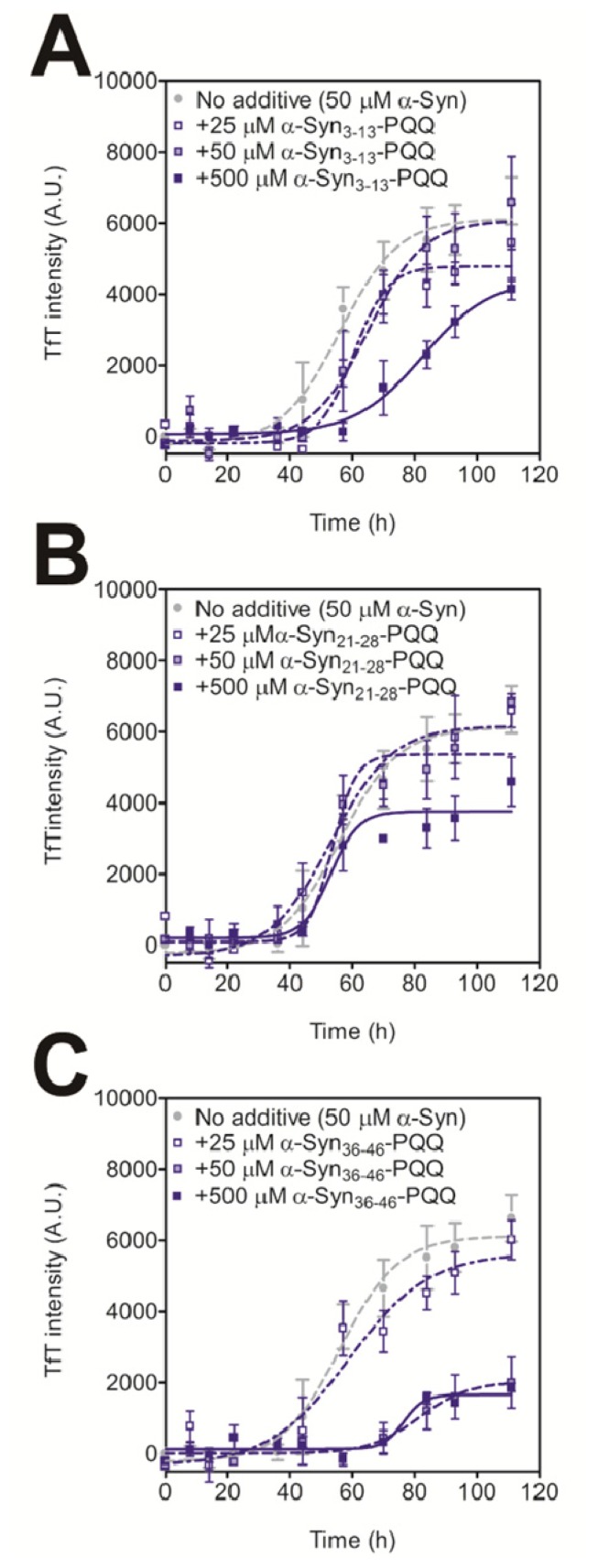
Inhibitory effect of PQQ-modified α-Syn peptides on the fibril formation of α-Syn. The time course of amyloid fibril formation by α-Syn was determined by the TfT assay. The sigmoidal curve analysis was performed by PRI. The fibril formation of 50 μM α-Syn in the presence or absence of 25, 50, and 500 μM the α-Syn_3–13_-PQQ (**A**), α-Syn_21–28_-PQQ (**B**), and α-Syn_36–46_-PQQ (**C**) were analyzed (*n* = 3).

In order to evaluate the inhibitory activity of PQQ-modified peptides on amyloid formation of intact α-Syn, we carried out a thioflavin T (TfT) assay [21]. We have previously reported that the amyloid fibril formation of 140 μM α-Syn was completely prevented by 70 μM PQQ [21]. The result indicates that half-equimolar amount of PQQ completely inhibits α-Syn fibril formation *in vitro*. Thus, we added 25 μM, 50 μM, and 500 μM of PQQ-modified peptides to 50 μM α-Syn. All the PQQ-modified peptides prevented intact α-Syn fibril formation, but their inhibitory effects were dependent on the peptide sequence (Figure 1). α-Syn_36–46_-PQQ showed higher inhibitory activity than α-Syn_3–13_-PQQ and α-Syn_21–28_-PQQ. It has been reported that amino acids 36–46 (GVLYVGSKTKE) are included in the core region of amyloid fibers of α-Syn [24–26], and Tyr39 residue has an important role in α-Syn fibril formation [27]. These results suggest that the PQQ-modified α-Syn_36–46_ specifically interacts with the core region of intact α-Syn to inhibit amyloid fibril formation.

We analyzed molecular mass of α-Syn_36–46_-PQQ by MALDI-TOF-MS. We detected three peaks at a molecular mass of 1180, 1492, and 2984 corresponding to unmodified peptide, one peptide modified with one PQQ and two peptides modified with two PQQ, respectively (Figure S3). These data indicated that PQQ-modified peptide is formed at a molar ratio of 1:1. The stoichiometry of modification is also supported by size exclusion chromatography purification of α-Syn_36–46_-PQQ, because we detected only one peak that contains PQQ-modified peptide.

**Figure 2:**
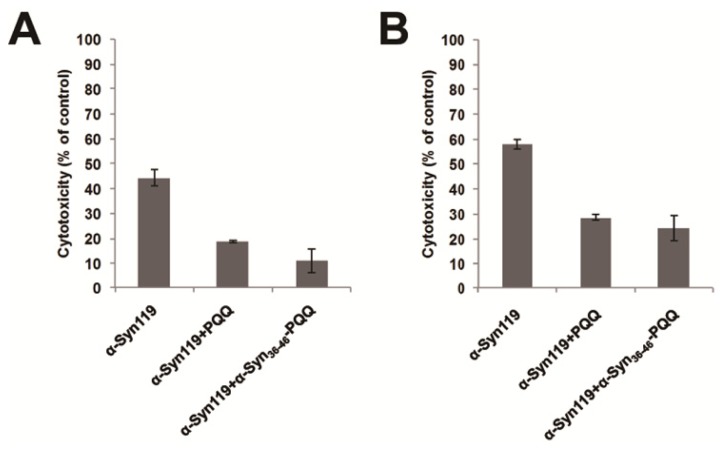
Cytotoxicity evaluation of α-Syn119 aggregates incubated with α-Syn_36–46_-PQQ. In the presence or absence of inhibitors, α-Syn119 samples were incubated for 18 h and then the cytotoxicity of the samples was analyzed by CC8 (**A**) and ATP assay (**B**). PQQ and α-Syn_36–46_-PQQ showed lower cytotoxicity than that of α-Syn119 (*p* < 0.0014 and *p* < 0.0028 in CC8 assay, respectively and *p* < 0.001 and *p* < 0.0063 in ATP assay, respectively).

Cytotoxicity of amyloid forming protein represents the presence of water soluble oligomer structure, which is the precursor of amyloid fibril. Therefore, we evaluated the cytotoxicity of α-Syn aggregates incubated with α-Syn_36–46_-PQQ by means of two different assays. In these assays, we utilized C-terminal truncated α-Syn (α-Syn119), which shows higher cytotoxicity than full-length α-Syn. We incubated α-Syn119 for 18 h in the presence or absence of α-Syn_36–46_-PQQ, and then U2-OS cells were exposed to the α-Syn119 samples for 48 h. The cell viability was measured by both of Cell Counting Kit-8 (CC8 assay) and CellTiter-Glo Luminescent Cell Viability Assay (ATP assay). These results indicated that α-Syn119 aggregates incubated with α-Syn_36–46_-PQQ showed lower cytotoxicity than that of α-Syn119 (Figure 2). Therefore, the cytotoxicity assays suggested that α-Syn_36–46_-PQQ inhibits the formation of cytotoxic oligomer formation of α-Syn.

### 2.3. Evaluation of Specificity of PQQ-Modified α-Syn_36–46_ Peptide

**Figure 3:**
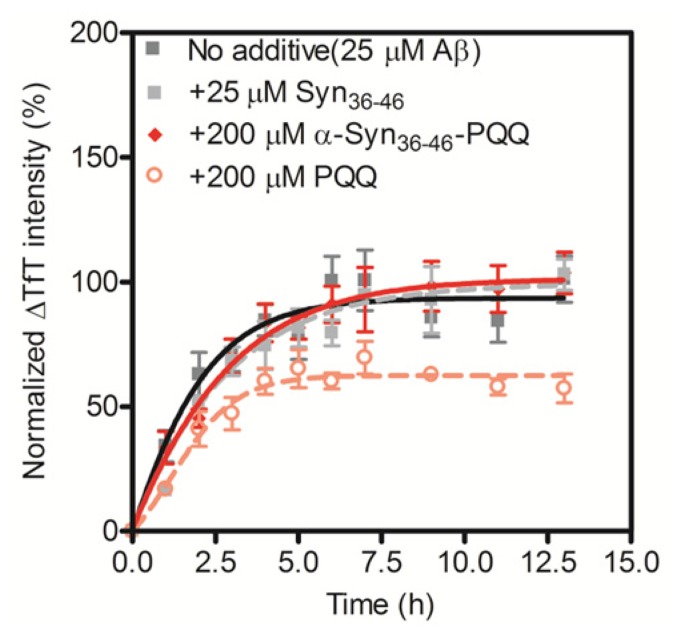
Inhibitory effect of α-Syn_36–46_-PQQ on the fibril formation of Aβ_1–42_. The time course of amyloid fibril formation of Aβ_1–42_ was determined using the TfT assay. The sigmoidal curve analysis was performed by PRI. The fibril formation of 25 μM Aβ_1–42_ in the presence of 25 μM unmodified α-Syn_36–46_, 200 μM α-Syn_36–46_-PQQ or 200 μM PQQ were analyzed (*n* = 3).

The grand average of hydropathy (GRAVY) value of α-Syn_36–46_ peptide is −0.245 [28], indicating that the peptide is hydrophilic. In the process of amyloid fibril formation, hydrophobic interactions play an important role. Thus, we assumed that the PQQ-modified α-Syn_36–46_ peptide would not interact with other amyloid-forming proteins. We carried out the TfT assay for Aβ_1–42_ in the presence of the α-Syn_36–46_-PQQ. We first confirmed that PQQ inhibited the amyloid formation of Aβ_1–42_, as we had reported previously (Figure 3). On the other hand, α-Syn_36–46_-PQQ did not inhibit nor accelerate the amyloid formation of Aβ_1–42_. These results suggest that α-Syn_36–46_-PQQ specifically inhibits the fibril formation of α-Syn.

### 2.4. Evaluation of Inhibitory Effects of Baicalein or EGCG-Modified α-Syn_36–46_ Peptide on Amyloid Formation of α-Synuclein

To investigate whether the other inhibitor-modified α-Syn_36–46_ would inhibit the amyloid formation of intact α-Syn, we prepared Baicalein or EGCG-modified α-Syn_36–46_ peptide. Baicalein and EGCG bind to Lys residues *via* Schiff-base formation as well as PQQ; thus, we assumed that Baicalein and EGCG-modified α-Syn_36–46_ would work as α-Syn inhibitors. The α-Syn_36–46_ peptide was incubated with Baicalein or EGCG for 14 days, and then Baicalein or EGCG modified peptide were separated by performing size exclusion chromatography. Baicalein and EGCG showed absorbance at 280 nm (Figure S4); thus, the elution was monitored by determining the absorbance at 280 nm. The Baicalein-modified sample and the EGCG-modified sample showed two peaks in absorbance at 280 nm, suggesting that there are two kinds of Baicalein or EGCG-modified α-Syn_36–46_. α-Syn_36–46_ peptide has two Lys residues that are capable to form Schiff-base formation. Thus, we assumed that the first peak contained one α-Syn_36–46_ peptide modified with two compounds and the second peak contained one α-Syn_36–46_ peptide modified with one compound. Following peak collection, we designated the first and second peaks of Baicalein-modified α-Syn_36–46_ and EGCG-modified α-Sy_n36–46_ as α-Syn_36–46_-Baicalein_P1, α-Syn_36–46_-Baicalein_P2, α-Syn_36–46_-EGCG_P1, and α-Syn_36–46_-EGCG_P2, respectively.

**Figure 4:**
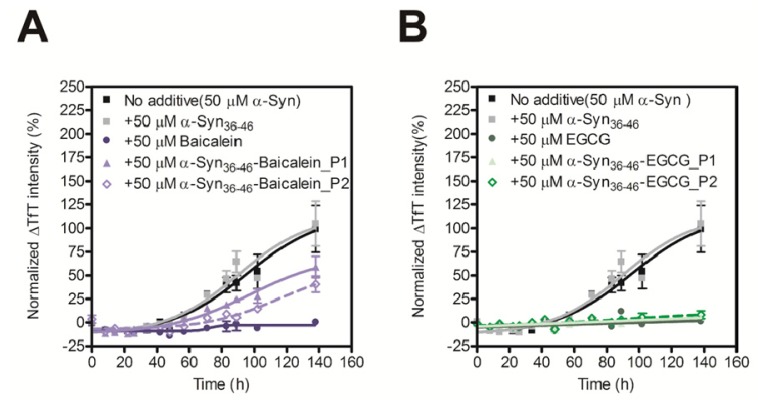
Inhibitory effect of α-Syn_36–46_-Baicalein (**A**) and α-Syn_36–46_-EGCG (**B**) on the fibril formation of α-Syn. The time course of amyloid fibril formation of α-Syn was determined using the TfT assay. The sigmoidal curve analysis was performed by PRI. The fibril formation of 50 μM α-Syn in the presence of 50 μM Baicalein, Baicalein-modified α-Syn peptides, EGCG or EGCG-modified α-Syn peptides were analyzed (*n* = 3).

In order to evaluate the inhibitory activity of the Baicalein or EGCG-modified α-Syn_36–46_ peptides on amyloid formation of α-Syn, we carried out the TfT assay. Although the unmodified α-Syn_36–46_ did not prevent amyloid formation of α-Syn, the Baicalein or EGCG-modified α-Syn_36–46_ peptides prevent amyloid formation of intact α-Syn (Figure 4). These results suggest that various α-Syn-specific inhibitors could be developed by modifying the α-Syn_36–46_ peptide with various inhibitors of fibril formation.

The inhibitory effect of amyloid formation in the presence of quinone molecules such as PQQ, EGCG and Baicalein is currently understood that the formation of quinone adducts of the parental amyloid forming proteins inhibits the further protein-protein oligomeric complex formation. These quinone molecules were bound with peptide by forming Schiff-base to produce PQQ, EGCG or Baicalein adducts. Schiff-base formation between α-Syn and PQQ or Baicalein was occurred at the Lys residues in the α-Syn molecule. Although our current investigation elucidated that, a particular partial peptide sequence, α-Syn36–46, was the most prone and significantly contributed in the inhibiting amyloid formation, other Lys residues within α-Syn molecule may be modified by PQQ via Schiff-base formation during the incubation with intact quinones. It was also suggested that with increasing the number of Lys residues in the amyloid forming proteins, the amyloid inhibitory effect by PQQ increased [22]. Therefore, the intact PQQ, EGCG or Baicalein binds with α-Syn at various position of Lys residues, consequently may coordinately show more strong inhibitory effect on amyloid formation than a PQQ or Baicalen adduct partial peptide sequence. In addition, the impact of the presence of intact PQQ or Baicalein is different, and Baicalein has been reported more effectively inhibiting α-Syn fibril formation [11]. The difference may be the sum of the ability of Schiff-base formation and the inhibitory effect of the adducts. However, the intact quinone molecules can easily form adducts *via* Schiff-base formation with any amine group. Although the inhibitor molecule modified peptide showed lower inhibitory effect compared with the intact one, target amyloid forming protein specific inhibition will be achieved using the strategy presented in this study.

## 3. Experimental Section

### 3.1. Preparation of Recombinant α-Synuclein

Human wild-type α-Syn was expressed in the *E. coli* BL21 (DE3) cell line transfected with the pET28a(+)/α-Syn plasmid and purified, as we reported previously [21].

### 3.2. LC/MS Analysis of Proteolytic-Digested α-Synuclein

The proteolytic-digested α-Syn was prepared as described previously [23]. Briefly, 4 mg/mL α-Syn was digested by endoproteinase Glu-C (Sigma, St. Louis, MO, USA) in PBS buffer (8.1 mM Na_2_HPO_4_, 1.4 mM KH_2_PO_4_, 137 mM NaCl, 2.7 mM KCl, pH 7.3) at 37 °C for 18 h, and then the undigested α-Syn and Glu-C were removed from the proteolytic mixtures by ultrafiltration through Amicon Ultra-4 filters (MWCO, 5000 Da; Millipore, Billerica, MA, USA). The proteolytic-digested α-Syn was subjected to LC/MS analysis (JMS-T100LC AccuTOF, JEOL, Akishima, Tokyo, Japan). The data were analyzed by ExPASyFindPept tool [29,30].

### 3.3. PQQ, Baicalein, EGCG-Modification of α-Syn Partial Peptides

The 280 μM α-Syn partial peptide (GenScript, Piscataway, NJ, USA) and 2.8 mM PQQ (Mitsubishi Gas Chemical, Chiyoda-ku, Tokyo, Japan), Baicalein or EGCG (Tokyo Chemical Industry, Chuo-ku, Tokyo, Japan) were co-incubated in PBS buffer. The sample containing peptide with PQQ was incubated at 37 °C with shaking for 7 days, and the sample containing peptide with Baicalein or epigallocatechin gallate (EGCG) was incubated at 37 °C with shaking for 14 days. The samples were loaded onto a PD-10 column (GE Healthcare, Chalfont St Giles, Buckinghamshire, UK) to remove the intact PQQ, Baicalein or EGCG. Then the eluted fractions were subjected to size exclusion chromatography (Superdex Peptide 10/300 GL, GE Healthcare, Chalfont St Giles, Buckinghamshire, UK) and eluted with a 30% acetonitrile in buffer containing 0.1% trifluoroacetic acid (TFA). The elution was monitored by determining the absorbance at 210 nm, 280 nm and 420 nm, simultaneously. The eluted fractions containing adduct were collected as PQQ, Baicalein or EGCG modified α-Syn partial peptides. The peptides were lyophilized and dissolved in PBS buffer before use. For MALDI-TOF MS analysis, α-Syn_36–46_-PQQ was dissolved in sinapinic acid matrix solution (70% acetonitrile, 0.1% TFA, 0.5 mg/mL sinapinic acid) and then analyzed by MALDI-TOF MS spectrometer Voyager-DE STR (Applied Biosystems, Carlsbad, CA, USA).

### 3.4. Amyloid Fibril Formation Analysis

Purified α-Syn was ultracentrifuged (150,000*g*, 1 h, 4 °C) to remove any aggregates. Each peptide was mixed with 1.0 mg/mL (70 μM) α-Syn in PBS buffer containing 0.02% NaN_3_ as an antiseptic agent. For each sample, 250 μL were aliquoted in triplicate into a 96-well microtiter plate together with a Teflon ball. The plates were covered with a seal and incubated at 37 °C with shaking at 700 rpm. Amyloid formation was monitored by the TfT assay. Aliquots of 2.5 μL were removed from the incubated sample and added to 250 μL of 25 μM TfT in PBS buffer. TfT fluorescence was recorded at 486 nm with excitation at 450 nm using an ARVO MX 1420 multilabel counter (PerkinElmer, Waltham, MA, USA).

For analysis of Aβ_1–42_ fibril formation, the purchased human Aβ_1–42_ (trifluoroacetate form, Peptide Institute, Minoh-shi, Osaka, Japan) was prepared as described previously [31]. The 25 μM Aβ_1–42_ was mixed with 200 μM PQQ or 200 μM PQQ-modified α-Syn peptide in PBS buffer with 0.02% NaN_3_ and then incubated at 37 °C in a 0.5 mL tube. Aliquots of 10 μL were removed from the incubated sample and TfT fluorescence was recorded as described above.

### 3.5. Cytotoxicity Assay of α-Synuclein

Cytotoxicity assay of α-Synuclein was carried out based on two different commercially available methods. Briefly, 100 μM of α-Syn119 was incubated for 18 h in the presence or absence of 200 μM of PQQ or α-Syn_36–46_-PQQ. The incubated samples (final concentration of α-Syn119 was 10 μM) were added to U2-OS cells and the cells were exposed to the α-Syn119 samples for 48 h. The number of viable cells was monitored by both of the Cell Counting Kit-8 (CC8 assay; Dojindo Molecular Technologies, Inc., Kamimashiki, Kumamoto, Japan) and CellTiter-Glo Luminescent Cell Viability Assay Kit obtained from Promega (ATP assay; Madison, WI, USA), according to the manufacturer’s protocol.

## 4. Conclusions

In this study, we demonstrate that the PQQ-modified α-Syn_36–46_ peptide, which is a partial sequence of α-Syn, prevented α-Syn amyloid fibril formation, but did not inhibit Aβ_1–42_ fibril formation. We also observed that other quinone inhibitors, such as Baicalein and EGCG, modified α-Syn_36–46_ peptide work as fibril formation inhibitors for α-Syn. Therefore, the α-Syn_36–46_ peptide will be a platform for the development of α-Syn-specific inhibitors that are modified with amyloid fibril formation antagonists. Our study provides proof-of-concept that combining specific peptide sequences with various quinone compounds is a novel strategy for the development of plaque type-specific amyloid formation inhibitors.

## Figures and Tables

**Table 1:** Identified peptide sequences.

Position	Sequence
3–13	VFMKGLSKAKE
21–28	KTKQGVAE
36–46	GVLYVGSKTKE
47–57	GVVHGVATVAE
127–131	MPSEE

## Supplementary Files

Supplementary File 1 Supplementary Information (PDF, 174 KB)
