# Supplementary material for: Partial Peptide of α-Synuclein Modified with Small-Molecule Inhibitors Specifically Inhibits Amyloid Fibrillation of α-Synuclein
Source: Int J Mol Sci. 2013 Jan 28;14(2):2590–600. doi: 10.3390/ijms14022590 (PMC3588004; doi:10.3390/ijms14022590)
Supplement: Supplementary File 1 — Supplementary Information (PDF, 174 KB) [file ijms-14-02590-s001.pdf]

Supplementary Information

Figure S1. Total ion chromatogram of the proteolytic-digested α-synuclein partial peptide in the LC/MS. The peak 3 and 4 correspond to the Sp-7 fraction, which has inhibitory activity against α-synuclein fibril formation.

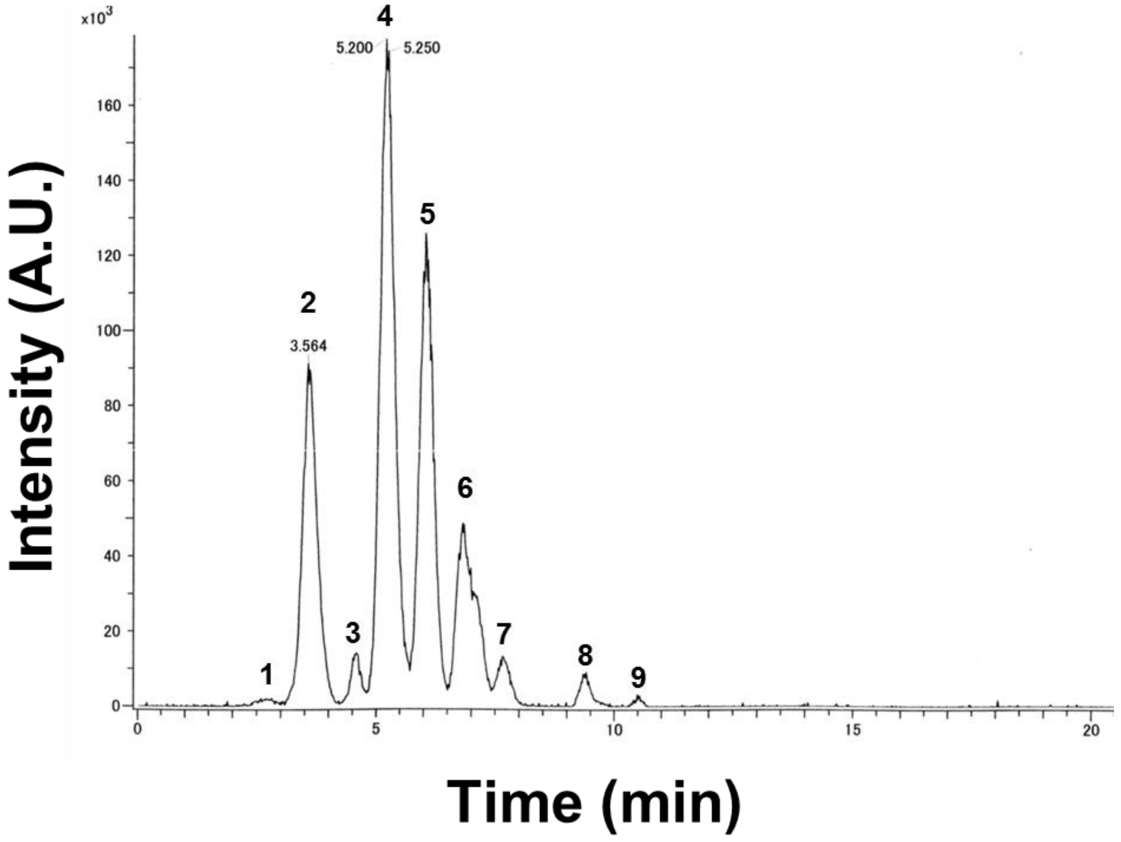

Figure S2. The size exclusion chromatography of PQQ-modified α-Syn_{3-13} (**A**), PQQ-modified α-Syn_{21-28} (**B**) and PQQ-modified α-Syn_{36-46} (**C**). In all case, the first peaks that eluted at 10 to 20 ml were corrected.

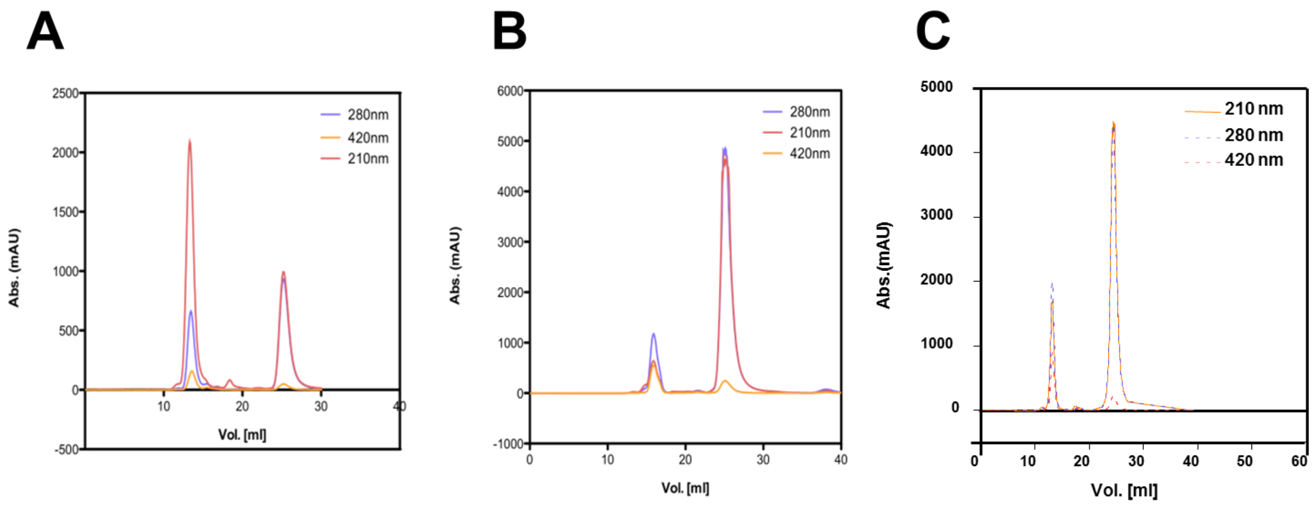

Figure S3. MALDI-TOF MS analysis of α-$Syn_{36\text{-}46}$-PQQ. Three peaks at a molecular mass of 1180, 1492, and 2984 correspond to unmodified peptide, one peptide modified with one PQQ and two peptides modified with two PQQ, respectively.

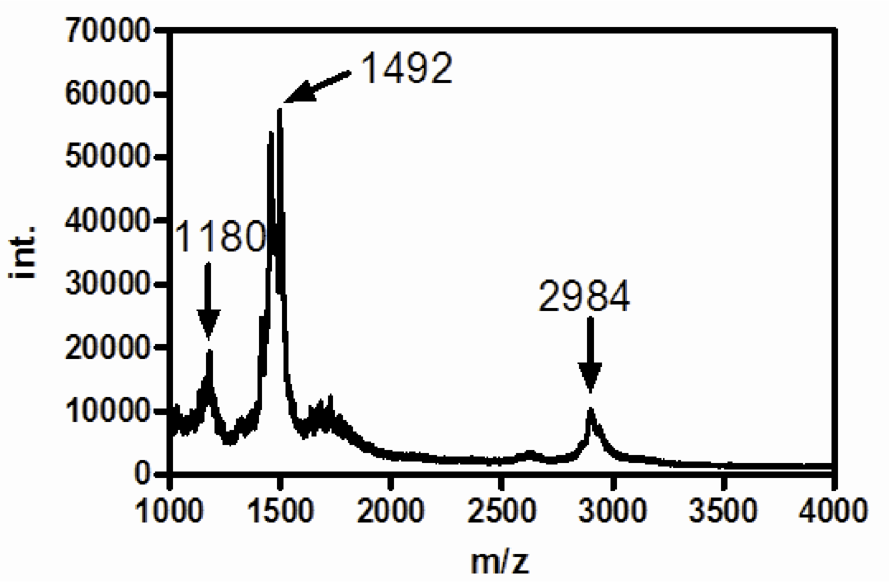

Figure S4. The size exclusion chromatography of Baicalein-modified α-$Syn_{36\text{-}46}$ (**A**) and EGCG-modified α-$Syn_{36\text{-}46}$ (**B**). In the both cases, 2 peaks were observed when the elution was monitored by determining the absorbance at 280 nm. The first and second peaks are highlighted in green and yellow, respectively.

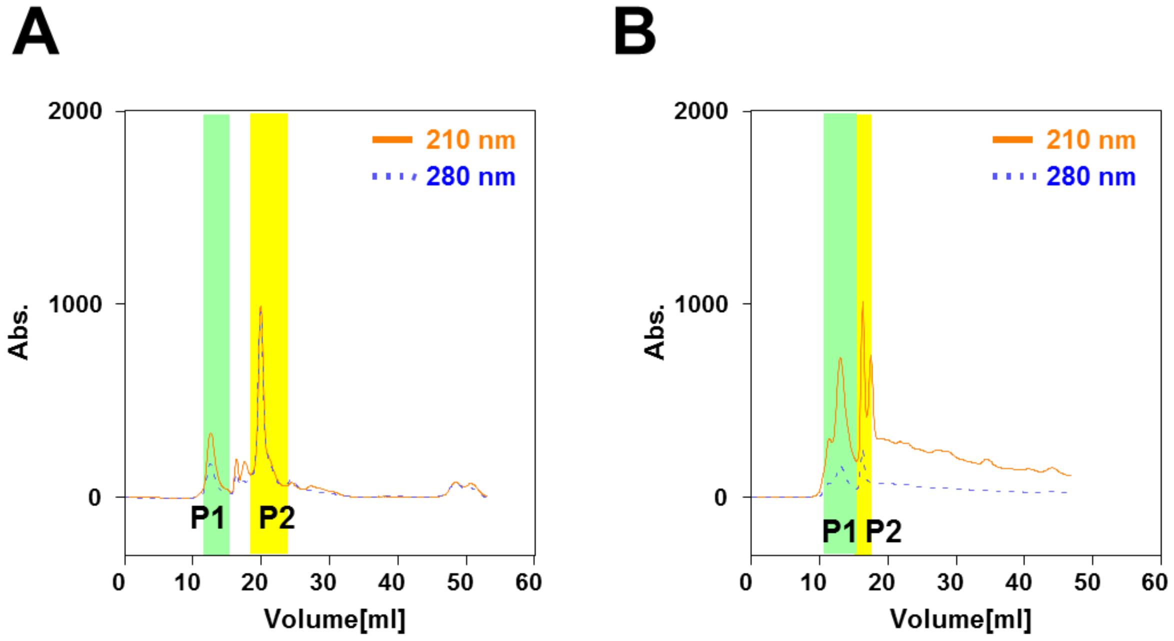

© 2013 by the authors; licensee MDPI, Basel, Switzerland. This article is an open access article distributed under the terms and conditions of the Creative Commons Attribution license (http://creativecommons.org/licenses/by/3.0/).
